# The Association Between Pica and Iron-Deficiency Anemia: A Scoping Review

**DOI:** 10.7759/cureus.37904

**Published:** 2023-04-20

**Authors:** Pallavi R Ganesan, Audrey A Vasauskas

**Affiliations:** 1 Department of Research, Alabama College of Osteopathic Medicine, Dothan, USA

**Keywords:** lithophagia, pagophagia, geophagia, iron deficiency, anemia, pica

## Abstract

There are many presentations of iron-deficiency anemia associated with pica in adults, but there is a lack of literature summarizing its different presentations. In this scoping review, we sought to identify the various presentations and if treatment of iron-deficiency anemia resolved the presenting symptoms of pica.

This review was conducted by completing the Preferred Reporting Items for Systematic Review and Meta-Analysis extension for Scoping Reviews (PRISMA-Scr) checklist. The following electronic databases were searched for potentially eligible articles: PubMed, ProQuest, and Bielefeld Academic Search Engine (BASE). Study screening procedures were completed with a narrative synthesis. The data is synthesized and interpreted by sifting, charting, and sorting based on organ systems. Twenty articles met the inclusion criteria and were included in the scoping review.

Regardless of other clinical presentations, the identification of pica symptoms allowed treatment for iron deficiency and led to the resolution of all symptoms in all 20 articles. Therefore, it is imperative to map the available evidence to inform clinicians and allow for better patient care.

## Introduction and background

The definition of pica, according to the Diagnostic and Statistical Manual of Mental Disorders, fifth edition (DSM-5), is the action of ingestion of non-nutritive substances, which is repeated for a period of at least a month and is developmentally inappropriate. Pica can have different names according to the substance eaten. There are many different non-nutritious items consumed: Geophagia is the consumption of earth, pagophagia is the consumption of a tray of ice, rhizophagy is the consumption of uncooked rice, and amylophagia is the consumption of starch [1]. There are many other unusual types of pica that have been reported, such as mothballs, egg shells, paper, cardboard, and others [2].

Pagophagia appears to be common in the United States of America (USA), affecting 25% of iron-deficient patients, while geophagia is more common in the rest of the world [1]. Geophagia is accepted in some cultures, especially in Africa [1]. Individuals are often reluctant to disclose or discuss unusual cravings and ingestion of abnormal substances with their physicians. In a study by Young et al. [3], many anthropologies, geographers, nutritionists, and doctors attempted to understand the basis for the cravings. There were two adaptive hypotheses formulated: (1) nutrient deficiency according to which calcium, sodium, zinc, and iron are ingested by geophagia to compensate for the lack and (2) protection from enterotoxin and parasites by reducing the permeability of the gut wall or binding to the toxin. However, there is no definite conclusion on the cause of pica in patients [3]. The side effects of pica include abdominal problems, tooth damage, and electrolyte disturbances.

In a study by Barton et al. [4], ice was the most common pica item (87.3% out of 103 patients reported). Cardiopulmonary symptoms and epithelial manifestations were the more common in patients who reported pica. All patients in this study with pica reported that their pica resolved within three weeks after administration of their first IV iron infusion.

Pica and iron deficiency

A patient requires the following general criteria to be diagnosed with iron-deficiency anemia (IDA) according to the World Health Organization: blood hemoglobin values of less than 7.7 mmol/l (13 g/dl) in men and 7.4 mmol/l (12 g/dl) in women. Clinical symptoms include fatigue, shortness of breath, pallor, alopecia, glossitis, pica, decreased cognitive abilities, attention, and concentration. Physicians use serum ferritin as the most reliable test for diagnosing absolute ID [5]. With IDA, it is important to identify the primary cause of the anemia and initiate treatment. The key causes of ID/IDA include decreased intake/absorption (dietary restrictions, Celiac disease, gastric bypass, gastroesophageal reflux disorder, *Helicobacter pylori* infection), iron sequestration (inflammatory diseases, chronic heart failure, chronic kidney disease, etc.), increase in iron demand in physiologic states (pregnancy, childhood, extreme exercises), and blood loss (vaginal, gastrointestinal loss, genito-urinary loss, blood donations, etc.) [4].

There have been many reports such as case studies and clinical studies, which discuss the association between pica and iron deficiency. According to Miao et al. [5], there is a reported frequency of 11% of iron-deficient patients with pica symptoms. In a meta-analysis by Miao et al. [5] which looked at 83 studies with 6,407 individuals with pica and 10,277 controls in total, it was reported that pica had an association of 2.35 times greater odds of anemia and lower zinc concentrations. Some authors have suggested that pica may be inducing iron deficiency by replacing dietary iron sources or inhibiting the absorption of iron [6]. However, in another study by Seim et al. [7], there is a suggestion that geophagia substances do not bind to bioavailable iron and are not responsible for reduced iron absorption. Many researchers and clinicians such as pediatricians and hematologists believe that iron deficiency itself induces pica. In the study by Young et al. [3], they examined the nutrient-deficiency hypothesis. This hypothesis states that geophagy occurs to compensate for the lack of iron, zinc, or calcium; thus, people with the greatest needs would practice geophagy more often. In this study, they excluded this hypothesis by proving that there was no correlation between the age when calcium and zinc were most needed and the prevalence of geophagy. Young et al. [3] found a statistically significant correlation between geophagia and anemia, but the geophagia did not seem to be aimed at correcting it.

In many of the case studies discussed in this scoping review, the patients were able to stop the craving for non-nutrients with iron treatment by days 5-8 of initiating iron therapy [8]. Auerbach and Adamson reported incidents of near-instantaneous elimination of pagophagia during IV infusions of iron. The correlation between serum iron levels and symptoms of pagophagia was best demonstrated by the disappearance of symptoms in 22 out of 23 patients with the symptoms of pagophagia or IDA [9]. The treatment modalities commonly used for iron treatment include red blood cell transfusions, oral iron supplementations (e.g., ferrous ascorbate, ferrous succinate), or IV iron transfusion [10].

In this scoping review, all case studies or clinical studies within the past 10 years which discussed the different systemic manifestations seen with pica symptoms and iron-deficiency symptoms are listed. The goal of this article is to encourage physicians to discuss abnormal eating habits during visits to highlight that pica is a common manifestation of IDA.

## Review

Materials and methods

To provide the outline of the prevalence of pica and IDA, an extensive literature search was carried out to explore the different manifestations within studies published in the past 10 years. The protocol was drafted using the Preferred Reporting Items for Systematic Reviews and Meta-Analysis extension for Scoping Reviews (PRISMA-Scr) checklist.

Search strategy

The literature search strategy targeted human studies published in English which were available between January 1, 2011, and December 31, 2021. The following electronic databases were reviewed: PubMed, ProQuest, and Bielefeld Academic Search Engine (BASE). The study records were identified based on the following group of search terms: (1) “pica” AND “iron-deficiency anemia.” Descriptive information was extracted, and the outcomes were categorized according to organ systems. The literature search was conducted on January 15, 2022. The final search results were exported into EndNote, and duplicates were removed by a single reviewer. The search strategy is summarized in Table 1.

**Table 1:** Literature search strategy

Search Engine	PubMed
Keywords	(pica) AND (iron-deficiency anemia)
Species	Humans
Publication date	2011-2021
Publication type	Case Reports, Reviews

Inclusion and exclusion criteria

The scoping review included peer-reviewed studies if they had “iron-deficiency anemia” and “pica” as presenting symptoms and were published between 2011 and 2021. The review included the many forms of pica such as desiderosmia, coprophagia, geophagia, etc.

Articles were excluded if they were not published in English, had no full text availability, conducted non-human research, or were conference reports, study protocols, or systematic reviews.

Screening and data charting

Titles and abstracts were screened for relevance, and eligible articles were evaluated based on full-text articles by a single reviewer. For each article included in the review, citation information was exported into EndNote and was manually abstracted by a single reviewer. No quality appraisal was taken for this scoping review as the goal is to summarize the existing evidence on the topic to inform future research, clinical practice, and education, not to include or exclude studies based on their quality.

Synthesis of results

The case studies were grouped by the organ system affected and the presenting symptoms. The studies were then summarized based on presenting symptoms, treatment modalities, and post-treatment results.

Results

When researching with ProQuest, there were 516 results within the past 10 years with the aforementioned keywords. Based on those searches with the inclusion and exclusion criteria, there were four articles that met the requirements.

In PubMed, there were 260 results with the aforementioned keywords, in which 14 articles met the selection criteria.

Using the BASE database, the same keywords were used with 104 results in the past 10 years. Four articles were out of the time range, and two articles were found that matched the keywords.

**Figure 1:**
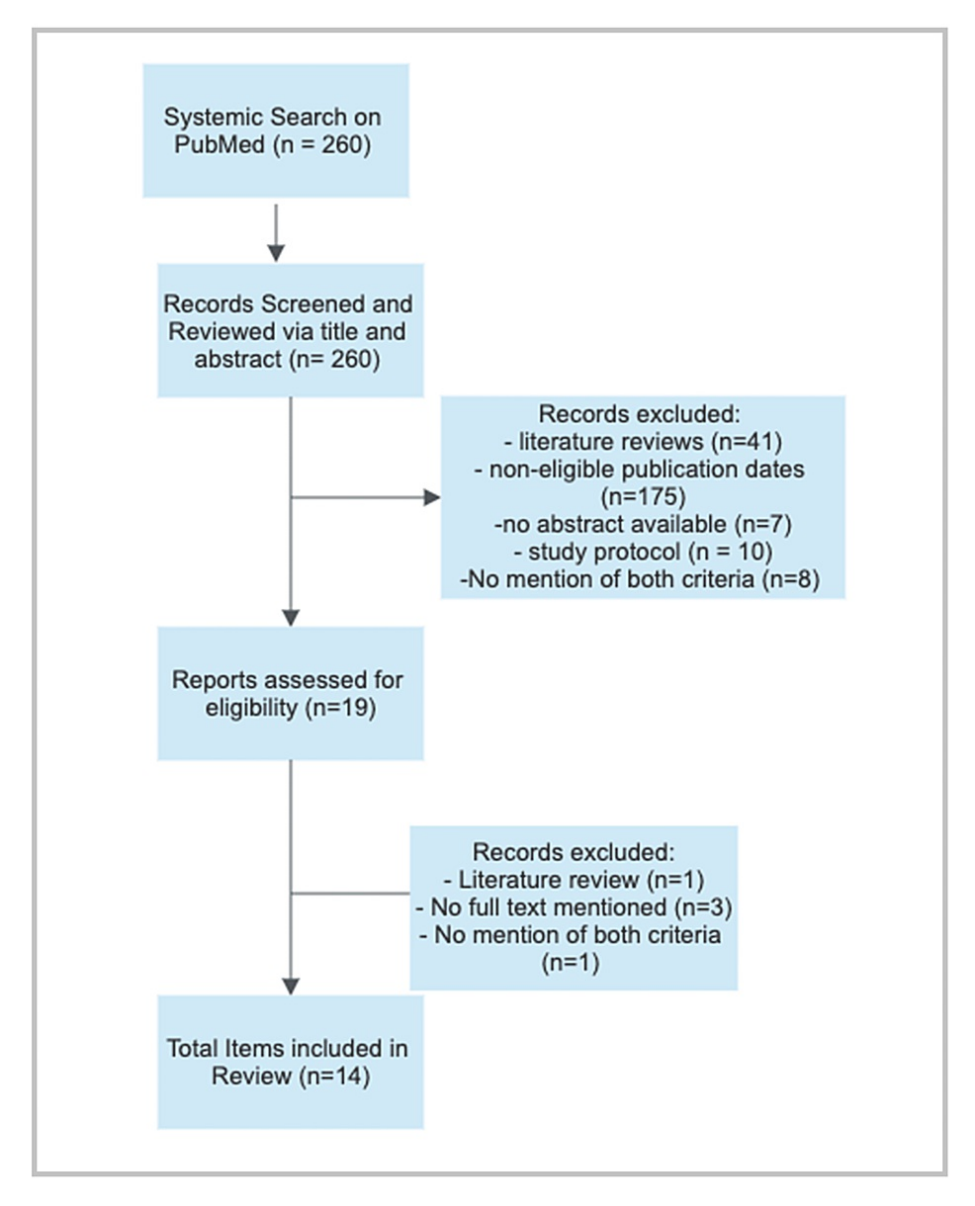
PubMed search strategy

**Figure 2:**
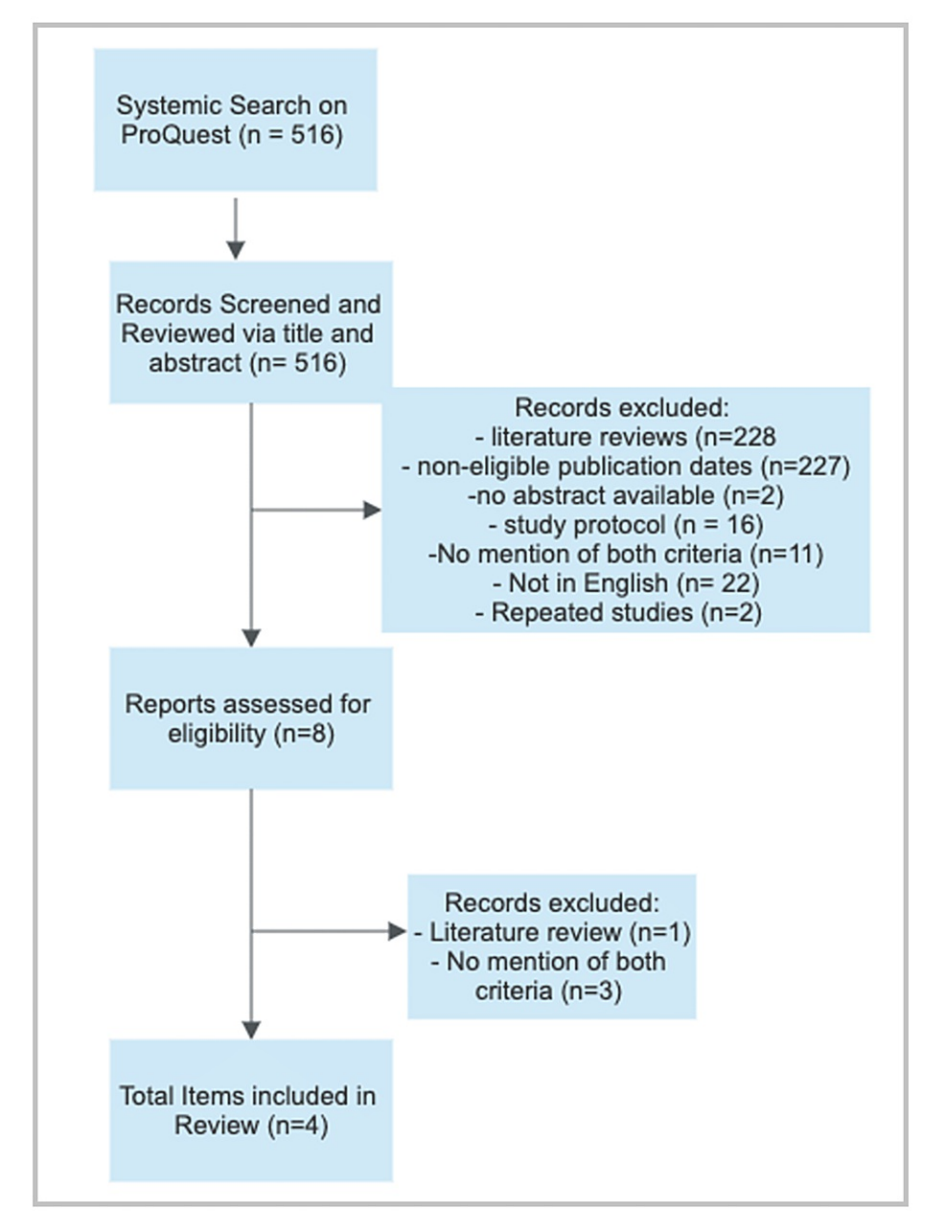
ProQuest search strategy

No relevant gray literature articles that met the criteria were discovered. The following gray literature sites were reviewed: CogPrints, MedNar, MetaLib, and OpenGrey. The same keywords were used to search these databases. The search procedures are outlined in Figures 1, 2.

A total of 20 articles were included in the review, of which six articles had neurological presentations, nine articles had gastrointestinal presentations, two had renal presentations, one had a pulmonary presentation, and two had other organ system presentations.

The articles are described in Table 2, together with the description of the clinical presentation, intervention, and outcomes. Overall, the body of evidence is comprised of different presentations of pica with weak pathological understanding.

**Table 2:** Case reviews CNS: Central nervous system; IDA: Iron-deficiency anemia; PDCB: Paradichlorobenzene; PBC: Primary biliary cholangitis; RYGBP: Roux-en-Y gastric bypass.

Authors	Article	Results	Source
Neurological			
Leong et al., 2020 [11]	Mothball ingestion as a manifestation of pica leading to paradichlorobenzene CNS toxicity	Patient after IDA treatment had improved iron symptoms and decreased PDCB toxicity and returned to normal state.	PubMed
Gruenstein et al., 2021 [12]	Trichotillomania due to pica in a 23-month-old patient with concomitant iron-deficiency anemia and lead poisoning	Pica and lead poisoning led to this patient's presentation, which resolved after lead chelators and iron medications were used.	PubMed
Acik, 2019 [13]	Recognizing the unusual findings: cases of desiderosmia	In all cases, patients lost the desire to smell exhaust, gasoline, or menthol after treatment.	PubMed
Sher and Maldonado, 2014 [14]	An insatiable desire for tofu: a case of restless legs and unusual pica in iron-deficiency anemia	After two months of iron-replacement therapy, the patient’s restless leg sensation was no longer felt.	PubMed
Sharma et al., 2011 [15]	Coprophagia and pica in individuals with mild to moderate dementia and mixed (iron deficiency and macrocytic) anemia	The patient on a therapeutic dose of psychiatric medications had improved symptoms of coprophagia after treatment with iron treatment.	PubMed
Bedanie et al., 2020 [16]	Pica/pagophagia-associated hyponatremia: patient presenting with seizure	The patient with ice pica presents with symptoms of hyponatremia and seizures. Treatment resolved the symptoms.	PubMed
Gastrointestinal			
Onuorah et al., 2019 [17]	Unusual presentation of pica in iron-deficiency anemia associated with primary biliary cholangitis	The patient with PBC and esophageal varices causing IDA and pica presentations improved clinically after iron replacement, banding of varices, and ursodeoxycholic acid.	PubMed
Kushner and Retelny, 2005 [18]	Emergence of pica accompanying iron-deficiency anemia after gastric bypass	Patients presented with pica after RYGBP in which iron infusion and iron medication caused diminished presentation of pica.	ProQuest
Tabaac and Tabaac, 2015 [19]	Pica patient, status post-gastric bypass, improves with change in medication regimen	A patient with a history of gastric bypass has reduced cravings for eating cardboard after treatment with iron, leading to reduced abdominal distension and pain and complete resolution of pica within six months of treatment.	Bielefeld Academic Search Engine (BASE)
Barton et al., 2016 [20]	Pica for uncooked basmati rice in two women with iron-deficiency anemia	Patients with a craving for eating uncooked rice presented with abdominal discomfort. Treatment resolved the symptoms.	PubMed
Cannalire et al., 2018 [21]	Rapunzel syndrome: infrequent cause of severe iron-deficiency anemia and abdominal pain in pediatric ER	The patient presents with gastric bezoars caused by severe iron-deficiency anemia. Treatment resolved the symptoms.	PubMed
Kurtz et al., 2020 [22]	Xylophagia: a rare form of pica	A patient presented with the craving of eating paper. Treatment resolved the symptoms.	PubMed
Nield et al., 2011 [23]	16-year-old boy with anemia, pica	A 16-year-old boy eating drywall presented with abdominal pain. Labs showed iron deficiency, and treatment improved anemia and pica. Celiac disease was identified because of pica.	ProQuest
Nakagami et al., 2021 [24]	Carotenemia induced by iron deficiency	The patient reported eating excessive carrots and mint candies and presented with fatigue and iron deficiency. Treatment of IDA resolved the compulsion to eat carrots and cadies and resolved carotenemia.	ProQuest
Singh et al., 2016 [25]	Atypical jejunal perforation due to cotton threads bezoar in postpartum woman associated with pica	A postpartum patient presented with jejunal perforation leading to the discovery of bezoar. Labs indicated anemia. Treatment of IDA reduced the symptoms.	PubMed
Pulmonary			
Ekinci et al., 2021 [26]	A rare complication of pica: stone aspiration with severe respiratory distress	A pediatric patient presents with stone aspiration because of pica leading to the discovery of IDA.	PubMed
Renal			
Rogers et al., 2017 [27]	Sodium chloride pica causing recurrent nephrolithiasis in a patient with IDA	An investigation led to the discovery of pica leading to increased salt intake, thus kidney stones. Treatment resolved further nephrolithiasis.	PubMed
Schmidt et al., 2021 [28]	A rare case of hypokalemia and hypophosphatemia secondary to geophagia	A patient with clay ingestion presented with hypokalemia and hypophosphatemia in which iron treatment resolved all electrolyte abnormalities.	ProQuest
Other organ systems			
Epler et al., 2017 [29]	Pica in pregnancy: an unusual presentation	A 37-week-gestational patient presented with gastritis and e*sophagitis.*	PubMed
Camli et al., 2014 [30]	Iron-deficiency anemia case that came with raw rice consumption	The patient’s symptoms of raw rice consumption improved after iron medication.	Bielefeld Academic Search Engine (BASE)

Some of the case studies are summarized below.

Neurological

There were six case studies that had neurological manifestations. In one case study, a patient presented with recurrent mothball ingestion that led to paradichlorobenzene central nervous system (CNS) toxicity and concomitant iron deficiency. She was treated with iron-replacement therapy and oral iron supplements and returned to her usual state of health in several months [11]. There was one case of trichotillomania due to pica with concomitant IDA [12]. The patient was started on iron supplementation. In a case study by Acik, different cases of patients with desiderosmia (desire to smell certain odors) that developed with iron deficiency were presented. All three cases were treated with iron supplementation [13]. Another neurological manifestation included a patient with restless leg syndrome and IDA with a desire to consume excess tofu [14]. A different patient also presented with coprophagia (desire to eat her own feces) due to her moderate dementia and concomitant IDA [15]. The patient was treated with iron supplementation and psychiatric medication and improved significantly. The last manifestation was a patient who presented with seizures due to pica-associated hyponatremia [16]. All patients who presented with neurological symptoms resolved all symptoms of pica with the treatment of iron deficiency.

Gastrointestinal

There were nine articles that had gastrointestinal presentations. In a study by Onuorah et al., a patient presented with symptoms of pica associated with primary biliary cholangitis discovered by CT imaging. Her symptoms of craving lemon were assumed to be causing her esophageal varices and biliary cholangitis [17]. In another case study, two patients presented with pica after their Roux-en-Y gastric bypass. Both patients had cravings for ice and had lab values associated with IDA [18]. In another article, another patient presented with a need for gastric bypass because of cardboard and paper bezoar causing gastric obstruction [19]. All the patients described in these cases had improved symptoms of pica after iron treatment and supplemental medications. All case studies with gastrointestinal presentations are included in Table 2 [20-25].

Pulmonary

There was one case study that had pulmonary manifestation. A pediatric patient presented with foreign body aspiration, and after urgent bronchoscopy, the patient was diagnosed with pica because of a compulsive need to eat coins. In this patient, after surgical intervention, iron treatment was initiated, and there was an improvement in the pica symptoms [26].

Renal

There were two renal presentations of IDA and pica. In one presentation, the patient presented with nephrolithiasis. After further investigation, the patient mentioned craving for ice and salt, which explained her nephrolithiasis [27]. In another renal presentation, a patient presented with hypokalemia and hypophosphatemia because of clay ingestion [28]. In these three patients, symptoms of pica resolved after iron treatment.

Other Organ Systems

In one case study by Epler et al., a 37-week gestational patient presented with esophagitis and gastritis secondary to laundry detergent consumption. In this patient, an iron infusion caused the resolution of her cravings [29]. In another case study, a patient with no other comorbidities presented with raw rice consumption leading to the discovery of IDA. Treatment resolved the symptoms [30].

Discussion

The prevalence of pica is varied greatly due to different diagnostic criteria, different methods, different studies, and different cultural practices. Pica is more common than expected but is often overlooked by physicians or underreported by patients [31]. In one study, results indicated that IDA can lead to the development of pica in some patients but not others, but there was no understanding of the dichotomy [3].

In this scoping review, 20 articles were identified addressing the different clinical manifestations of both pica and IDA, which were published between 2011 and 2021. Our findings indicate a paucity of research focusing on the pathophysiology of pica appearing as a presenting symptom. The nature of pica to often be considered an associated symptom rather than a presenting symptom makes it difficult for clinicians to use it as a clinical marker to indicate IDA.

The treatment of IDA is inexpensive compared to the medical costs of many of the more severe presenting symptoms seen in this review. If further research can be conducted on the pathophysiology of pica as a warning sign of severe iron deficiency, the burden on both the patient and the system can greatly be reduced by preventing the progression of disease severity.

Limitations

This scoping review has some limitations. To make this review more feasible to conduct, only three sources of databases were searched. Further, studies that did not result in the resolution of iron deficiency with iron treatment were not included. This could mean that other treatments and clinical decisions were not discussed in this review, which could create a challenge clinically. Another limitation of this study relates to the multiple presentations that occur with pica. A careful review was conducted to ensure that all types of presentations were included; however, due to the presentations ranging from mud to glass, there was a lack of scientific guidelines on what exactly constitutes pica.

Nevertheless, the strength of the study includes a strong development according to a predefined protocol, a systematic and transparent approach to the search of the studies, and reporting according to the PRISMA extension for scoping reviews.

## Conclusions

The wide range of clinical presentations of pica as a symptom of IDA in the population poses a challenge of diagnosis to physicians. Many physicians often do not ask patients specifically about signs of pica by addressing abnormal eating habits. However, by discussing pica more frequently, patients can be treated much earlier and prevent serious hospitalization.

Many of these case reports show that many non-developed areas of the world are affected greater, most likely due to challenges in access to healthcare, differences in public health efforts, and possibly social stigma. Further research is encouraged to evaluate the cultural and regional differences between pica presentations. Overall, the aim of the scoping review was to group different pica manifestations and help identify the gaps in the literature to hopefully guide a future systematic review or original study to understand the pathological causes of pica.
